# Supplementary material for: Fetal bovine and calf serums differ in their contents of endocannabinoids, unsaturated fatty acids, monoacyl-glycerols, N-acyl-ethanolamines, oxylipins, and cytokines
Source: Front Immunol. 2026 Oct 16;17:1856768. doi: 10.3389/fimmu.2026.1856768 (PMC13719332; doi:10.3389/fimmu.2026.1856768)
Supplement: Supplementary file 1 [file SupplementaryFile1.docx]

**Supplementary Figure 1.** **Metabolites that were significantly different between FBS and CS, with a log₂FC between -1 and 1. A)** AA; **B)** 2-PG; **C)** DHEA; **D)** 12-HETE; and **E)** Cytokines/chemokines**. A-E)** Non-parametric Wilcoxon rank-sum test was performed, as most of the metabolites didn’t follow a normal distribution. Significance was set at *p*<0.05 (*), *p*<0.01 (**), and *p*<0.001 (***). Data were obtained by analyzing 18 FBS samples and 13 CS samples.

**Supplementary Figure 2.** Metabolites that were not significantly different between FBS and CS. **A)** Fatty Acids, **B)** 2-EPG, **C)** NAEs, **D)** PGF_2α_, **E)** lipoxygenase metabolites, **F)** MCP-1 (CCL2). **A-F)** Non-parametric Wilcoxon rank-sum test was performed, as most of the metabolites didn’t follow a normal distribution. Significance was set at *p*<0.05 (*). Data were obtained by analyzing 18 FBS samples and 13 CS samples.

**Supplementary Figure 3. Correlations between lipids and bovine cytokines.** Spearman correlations, FDR corrected. Significance was set at *p*<0.05 (*), *p*<0.01 (**), and *p*<0.001 (***). Data were obtained by analyzing 18 FBS samples and 13 CS samples.

## Supplementary Tables

**Supplementary Table 1. Serum samples utilized in this study.**

| Serum NAME | PROVIDER | CATALOG # | | LOT number |
| --- | --- | --- | --- | --- |
| FBS1 | Wisent | 080-150 | | 115698 |
| FBS2 | Gibco | 123483 | | 263590RP |
| FBS3 | Wisent | 080-105 | | 115710 |
| FBS4 | Wisent | 080-105 | | 115714 |
| FBS5 | Avantor | 97068-085 | | 228K22 |
| FBS6 | Cornings | 35-077-CV | | 22023001 |
| FBS7 | Wisent | 098-150 | | 115715 |
| FBS8 | Wisent | 098-150 | | 112733 |
| FBS9 | Wisent | 080-150 | | Unknown - 2019 |
| FBS10 | Wisent | 080-150 | | Unknown - 2021 |
| FBS11 | Cornings | 35-010-CF | | 80A12-2 |
| FBS12 | Gibco | A31607-01 | | C3022585RP |
| FBS13 | Gibco | A47668-01 | | U2825973RP |
| FBS15 | Cornings | 35-077-CV | | 22023001 |
| FBS16 | Wisent | 080-105 | | 115745 |
| FBS17 | Cornings | 35-077-CF | 15822002 | |
| FBS18 | Wisent | 098-105 | | 185747 |
| FBS19 | Gibco | A31607-01 | | 2635906RP |
| CS1 | Cytiva | SH30541.03 | | AG29548253 |
| CS2 | ATCC | 30-2030 | | 80207171 |
| CS3 | Wisent | 074-150 | | 87129 |
| CS5 | Gibco | 16170-078 | | 1847453 |
| CS7 | Thermofisher | SH30541.02 | | AYK170734 |
| CS8 | Gibco | 16030-074 | | 2657905 |
| CS9 | Gibco | 26010-066 | | 2453222 |
| CS10 | Gibco | 16010-167 | | 2526356 |
| CS11 | Wisent | 074-110 | | 87138 |
| CS12 | Wisent | 074-110 | | 87139 |
| CS13 | Wisent | 074-110 | | 87140 |
| CS14 | ATCC | 30-2030 | | 62262399 |
| CS15 | Gibco | 16170-086 | | 2402160 |

**Supplementary Table 2. Key analytical parameters utilized to quantitate lipid mediators.**

| **Analyte** | **Internal Standard** | **Ret. Time (min)** | **ESI** | ***m/z* Quantifier** | ***m/z* Qualifier** | **Q1 Pre Bias^a^ (V)** | **Collision energy (V)** | **Q3 Pre Bias (V)** | **LLOQ^b^ (fmol)** |
| --- | --- | --- | --- | --- | --- | --- | --- | --- | --- |
| 6-keto PGF_1α_ | PGE_2_-d4 | 3,52 | - | 369,30→163,10 | 369,30→245,15 | 20 | 26 | 13 | 500 |
| PGF_3α_ | PGE_2_-d4 | 3,99 | - | 351,30→193,30 | 351,30→307,40 | 18 | 22 | 11 | 500 |
| TXB_2_-d4 |  | 4,38 | - | 373,10→173,40 | 373,10→199,35 | 18 | 17 | 22 |  |
| TXB_2_ | TXB_2_ -d4 | 4,41 | - | 369,20→169,25 | 369,20→195,30 | 18 | 17 | 10 | 50 |
| PGE_3_ | PGE_2_-d4 | 4,20 | - | 349,30→269,35 | 349,30→313,35 | 18 | 16 | 21 | 50 |
| PGF_2α_ | PGE_2_-d4 | 4,45 | - | 353,40→309,30 | 353,40→192,95 | 14 | 19 | 10 | 50 |
| PGE_1_ | PGE_2_-d4 | 4,83 | - | 353,30→273,40 | 353,30→317,30 | 18 | 22 | 15 | 50 |
| PGE_2_-d4 |  | 4,69 | - | 355,20→275,35 | 355,20→319,35 | 20 | 17 | 30 |  |
| PGE_2_ | PGE_2_-d4 | 4,69 | - | 351,20→271,15 | 351,20→315,20 | 13 | 17 | 14 | 50 |
| PGD_2_ | PGE_2_-d4 | 4,91 | - | 351,30→271,20 | 351,30→233,10 | 10 | 16 | 14 | 50 |
| 1a,1b-dihomo-PGF_2α_ | PGE_2_-d4 | 5,49 | - | 381,20→337,40 | 381,20→319,40 | 20 | 22 | 14 | 50 |
| PGB_2_-d4 |  | 6,21 | - | 337,40→179,15 | 337,40→239,15 | 16 | 20 | 20 |  |
| 12-HHTrE | PGE_2_-d4 | 7,95 | - | 279,20→179,25 | 279,20→261,25 | 19 | 11 | 21 | 50 |
| PGF_2α_-EA | PGF_2α_-EA-d4 | 3,61 | + | 380,10→344,30 | 380,10→257,00 | -14 | -15 | -23 | 50 |
| PGF_2α_-EA-d4 |  | 3,61 | + | 384,00→348,20 | 384,00→348,20 | -15 | -19 | -25 |  |
| PGE_2_-EA-d4 |  | 3,69 | + | 382,10→364,35 | *-* | -14 | -15 | -24 |  |
| PGE_2_-EA | PGE_2_-EA-d4 | 3,70 | + | 378,10→360,25 | 378,10→342,25 | -26 | -14 | -24 | 50 |
| PGD_2_-EA | PGE_2_-EA-d4 | 3,88 | + | 378,10→360,25 | 378,10→342,25 | -14 | -13 | -11 | 50 |
| PGF_2α_-G | PGE_2_-G-d5 | 4,04 | - | 427,20→353,30 | 427,20→91,20 | 21 | 18 | 27 | 500 |
| PGE_2_-G-d5 |  | 4,18 | + | 414,00→396,30 | 414,00→299,25 | -29 | -13 | -18 |  |
| PGE_2_-G | PGE_2_-G-d5 | 4,21 | + | 444,25→391,20 | 444,25→409,30 | -20 | -15 | -25 | 50 |
| PGD_2_-G | PGE_2_-G-d5 | 4,37 | + | 444,25→391,30 | 444,25→409,30 | -21 | -16 | -26 | 50 |
| SDEA | EPEA-d4 | 9,17 | + | 320,20→62,15 | 320,20→91,10 | -24 | -22 | -23 | 50 |
| EPEA | EPEA-d4 | 10,15 | + | 346,20→62,20 | 346,20→91,10 | -16 | -18 | -24 | 50 |
| EPEA-d4 |  | 10,10 | + | 350,20→66,15 | 350,20→91,20 | -24 | -22 | -24 |  |
| LEA | LEA-d4 | 10,94 | + | 324,30→62,05 | 324,30→44,00 | -20 | -25 | -20 | 50 |
| LEA-d4 |  | 10,90 | + | 328,00→66,10 | 328,00→48,10 | -23 | -23 | -26 |  |
| DHEA | DHEA-d4 | 11,11 | + | 372,20→62,20 | 372,20→91,15 | -14 | -23 | -24 | 50 |
| DHEA-d4 |  | 11,06 | + | 376,30→66,00 | 376,30→91,10 | -14 | -22 | -23 |  |
| AEA-d4 |  | 11,08 | + | 352,10→66,20 | 352,10→91,20 | -13 | -22 | -24 |  |
| AEA-d8 |  | 11,04 | + | 356,40→44,15 | 356,40→63,25 | -17 | -22 | -15 |  |
| AEA | AEA-d4 | 11,11 | + | 348,30→62,25 | 348,30→287,30 | -16 | -23 | -23 | 50 |
| PEA | PEA-d4 | 11,44 | + | 300,30→62,10 | 300,30→44,05 | -26 | -20 | -23 | 50 |
| PEA-d4 |  | 11,42 | + | 304,00→62,30 | 304,00→44,10 | -22 | -22 | -23 |  |
| OEA | OEA-d4 | 12,07 | + | 326,00→62,05 | 326,00→44,10 | -18 | -23 | -20 | 50 |
| OEA-d4 |  | 12,03 | + | 330,20→66,10 | 330,20→48,10 | -23 | -22 | -24 |  |
| SEA | SEA-d3 | 13,15 | + | 328,20→62,10 | 328,20→44,00 | -22 | -22 | -23 | 50 |
| SEA-d3 |  | 13,14 | + | 331,10→62,20 | 331,10→44,10 | -12 | -22 | -24 |  |
| 1/2-SDG | 1-AG-d5 | 10,28 | + | 351,20→259,25 | 351,20→161,15 | -28 | -16 | -26 | 1000 |
| 1/2-EPG | 1-AG-d5 | 11,16 | + | 377,10→285,25 | 377,10→303,20 | -30 | -19 | -30 | 500 |
| 1/2-DHG | 1-AG-d5 | 11,98 | + | 403,20→311,20 | 403,20→293,20 | -15 | -19 | -19 | 500 |
| 1/2-LG | 1-AG-d5 | 11,90 | + | 355,10→263,15 | 355,10→245,25 | -26 | -15 | -27 | 5000 |
| 1-AG-d5 |  | 12,08 | + | 384,20→287,25 | 384,20→269,30 | -14 | -16 | -30 |  |
| 1/2-AG | 1-AG-d5 | 11,96 | + | 379,20→287,20 | 379,20→269,25 | -27 | -17 | -30 | 100 |
| 1/2-DPG(n-3) | 1-AG-d5 | 12,46 | + | 405,20→313,30 | 405,20→105,10 | -15 | -17 | -20 | 1000 |
| 1/2-OG | 1-AG-d5 | 12,99 | + | 357,20→265,40 | 357,20→247,40 | -10 | -14 | -17 | 1000 |
| 1/2-PG | 1-AG-d5 | 12,39 | + | 331,00→313,30 | 331,01→57,10 | -14 | -11 | -20 | 1000 |
| SDA | AA-d8 | 11,54 | - | 275,00→231,35 | 275,00→177,30 | 19 | 12 | 14 | 100 |
| DPEA (n-6) | DHEA-d4 | 12,04 | + | 374,20→62,10 | 374,20→91,15 | -29 | -22 | -25 | 50 |
| DPEA (n-3) | DHEA-d4 | 11,47 | + | 374,00→62,20 | 374,00→44,10 | -14 | -22 | -25 | 50 |
| EPA-d5 |  | 12,33 | - | 306,20→262,20 | - | 17 | 11 | 28 |  |
| EPA | EPA-d5 | 12,36 | - | 301,20→257,30 | 301,20→203,25 | 17 | 11 | 20 | 50 |
| DHA-d5 |  | 13,09 | - | 332,20→288,20 | - | 19 | 11 | 19 |  |
| DHA | DHA-d5 | 13,11 | - | 327,50→283,30 | 327,50→229,00 | 10 | 11 | 12 | 500 |
| AA-d8 |  | 13,18 | - | 311,20→267,25 | 311,20→182,85 | 16 | 13 | 11 |  |
| AA | AA-d8 | 13,22 | - | 303,20→259,30 | 303,20→205,15 | 8 | 13 | 11 | 50 |
| DPA-d5 |  | 13,33 | - | 334,40→290,35 | 334,40→236,40 | 18 | 14 | 16 |  |
| DPA (n-3) | DPA-d5 | 13,35 | - | 329,50→285,15 | 329,50→231,10 | 18 | 13 | 22 | 100 |
| DPA (n-6) | DPA-d5 | 13,45 | - | 329,10→285,35 | 329,10→231,30 | 17 | 12 | 12 | 50 |
| LA | AA-d8 | 13,23 | - | 279,20→261,40 | 279,20→58,90 | 17 | 17 | 15 | 50 |
| OA | AA-d8 | 13,58 | - | 281,20→263,45 | 281,20→97,00 | 15 | 19 | 20 | 50 |
| 15-HEPE-EA | 15-HEPE-EA-d4 | 7,01 | + | 344,10→62,20 | - | -12 | -22 | -22 | 50 |
| 15-HEPE-EA-d4 |  | 6,98 | + | 348,30→66,15 | 348,30→283,20 | -13 | -22 | -23 |  |
| 13-HODE-EA-d4 |  | 7,23 | + | 326,30→66,20 | 326,30→48,10 | -11 | -22 | -10 |  |
| 13-HODE-EA | 13-HODE-EA-d4 | 7,25 | + | 322,00→62,20 | 322,00→344,35 | -15 | -22 | -23 | 50 |
| 15-HETE-EA | 15-HEPE-EA-d4 | 7,71 | + | 346,40→62,25 | - | -26 | -22 | -25 | 50 |
| 15-HEPE-G-d5 |  | 7,85 | + | 380,10→283,20 | 380,10→257,05 | -28 | -18 | -30 |  |
| 15-HEPE-G | 15-HEPE-G-d5 | 7,88 | + | 375,20→283,20 | 375,20→131,10 | -25 | -16 | -30 | 50 |
| 12-HEPE-G | 15-HEPE-G-d5 | 8,05 | + | 375,30→283,25 | 375,30→265,30 | -15 | -17 | -29 | 100 |
| 13-HODE-G-d5 |  | 8,21 | + | 358,20→261,20 | 358,20→67,15 | -26 | -16 | -27 |  |
| 13-HODE-G | 13-HODE-G-d5 | 8,25 | + | 353,20→261,20 | - | -25 | -15 | -27 | 50 |
| 17-HDPA-EA | 15-HEPE-EA-d4 | 8,28 | + | 372,10→62,15 | 372,10→130,90 | -14 | -22 | -23 | 50 |
| 15-HETE-G | 15-HEPE-G-d5 | 8,59 | + | 377,25→285,20 | 377,25→267,20 | -15 | -15 | -17 | 50 |
| 17-HDHA-EA | 15-HEPE-EA-d4 | 8,71 | + | 370,20→62,10 | 370,20→309,25 | -27 | -22 | -25 | 500 |
| 17-HDHA-G | 15-HEPE-G-d5 | 8,76 | + | 401,20→309,35 | 401,20→129,05 | -14 | -15 | -20 | 50 |
| 17-HDPA-G | 15-HEPE-G-d5 | 9,19 | + | 403,10→131,15 | 403,10→311,25 | -19 | -36 | -21 | 100 |
| 18-HEPE | 15-HETE-d8 | 8,63 | - | 317,30→259,40 | 317,30→299,35 | 17 | 12 | 11 | 50 |
| 8,15-DiHETE | 15-HETE-d8 | 6,70 | - | 335,40→317,30 | 335,40→235,25 | 19 | 15 | 13 | 500 |
| 5,15-DiHETE | 15-HETE-d8 | 6,85 | - | 335,20→317,25 | 335,20→173,25 | 20 | 11 | 25 | 500 |
| 5,6-DiHETE | 15-HETE-d8 | 8,28 | - | 335,00→317,35 | 335,00→114,95 | 16 | 10 | 13 | 500 |
| 13-HOTrE | 13-HODE-d4 | 8,61 | - | 292,90→195,25 | 292,90→275,25 | 15 | 16 | 16 | 100 |
| 15-HEPE | 15-HETE-d8 | 8,97 | - | 317,40→219,25 | 317,40→255,30 | 18 | 12 | 17 | 50 |
| 12-HEPE | 15-HETE-d8 | 9,16 | - | 317,30→179,35 | 317,30→255,40 | 17 | 14 | 23 | 50 |
| 13-HODE-d4 |  | 9,34 | - | 299,10→198,15 | 299,10→217,15 | 16 | 18 | 16 |  |
| 12-HETE-d8 |  | 10,02 | - | 327,20→184,20 | 327,20→309,30 | 16 | 14 | 14 |  |
| 12-HETE | 12-HETE-d8 | 10,09 | - | 319,10→179,25 | 319,10→301,30 | 17 | 14 | 14 | 50 |
| 9-HODE | 13-HODE-d4 | 9,38 | - | 295,30→171,35 | 295,30→277,35 | 15 | 17 | 10 | 50 |
| 13-HODE | 13-HODE-d4 | 9,39 | - | 295,50→195,30 | 295,50→277,30 | 17 | 17 | 15 | 50 |
| 15-HETE-d8 |  | 9,64 | - | 327,20→226,20 | - | 22 | 14 | 14 |  |
| 15-HETE | 15-HETE-d8 | 6,73 | - | 319,40→219,30 | 319,40→301,25 | 17 | 12 | 17 | 50 |
| 13-KODE | 5-KETE-d7 | 9,76 | + | 295,20→277,30 | 295,20→179,25 | -20 | -10 | -17 | 50 |
| 15-KETE | 5-KETE-d7 | 10,07 | - | 317,20→113,00 | 317,20→273,35 | 15 | 16 | 14 | 50 |
| 11-HETE | 15-HETE-d8 | 9,92 | - | 319,30→167,35 | 319,30→149,20 | 17 | 15 | 21 | 50 |
| 8-HETE | 15-HETE-d8 | 9,99 | - | 319,30→155,25 | 319,30→301,35 | 17 | 15 | 12 | 50 |
| 17-HDHA | 15-HETE-d8 | 9,90 | - | 343,50→281,30 | 343,50→201,30 | 19 | 13 | 22 | 50 |
| 14-HDHA | 15-HETE-d8 | 10,06 | - | 343,50→281,30 | 343,50→161,20 | 19 | 13 | 16 | 50 |
| 7-HDHA | 15-HETE-d8 | 10,06 | - | 343,15→281,20 | 343,15→141,05 | 17 | 12 | 17 | 50 |
| 4-HDHA | 15-HETE-d8 | 10,43 | - | 343,15→281,15 | 343,15→101,00 | 17 | 12 | 17 | 100 |
| 12-KETE | 5-KETE-d7 | 10,39 | - | 317,10→273,35 | 317,10→153,20 | 10 | 13 | 12 | 100 |
| 5-HETE | 5-HETE-d8 | 10,15 | - | 319,30→115,05 | *-* | 17 | 15 | 26 |  |
| 5-HETE-d8 |  | 10,07 | - | 326,99→309,35 | 326,99→116,00 | 22 | 10 | 17 |  |
| 17-HDPA | 15-HETE-d8 | 10,34 | - | 345,20→327,10 | 345,20→247,35 | 17 | 13 | 24 | 500 |
| 17-oxo-DHA | 15-HETE-d8 | 10,27 | - | 341,20→297,20 | 341,20→111,05 | 18 | 12 | 22 | 1000 |
| 15-HETrE | 15-HETE-d8 | 10,27 | - | 321,50→303,40 | 321,50→221,30 | 18 | 14 | 13 | 100 |
| 12-HETrE | 15-HETE-d8 | 10,51 | - | 321,15→181,10 | 321,15→303,20 | 17 | 20 | 17 | 100 |
| 5-KETE | 5-KETE-d7 | 10,85 | - | 317,49→203,20 | 317,49→273,35 | 16 | 16 | 11 | 100 |
| 5-KETE-d7 |  | 10,78 | + | 326,20→194,20 | 326,20→308,30 | -21 | -14 | -19 |  |
| RVE1 | RVD2-d5 | 3,63 | - | 349,30→195,20 | 349,30→107,10 | 19 | 16 | 11 | 500 |
| RVD2-d5 |  | 4,93 | - | 380,40→175,20 | - | 20 | 23 | 15 |  |
| RVD3 | RVD2-d5 | 4,74 | - | 375,40→147,20 | 375,40→137,20 | 12 | 19 | 18 | 100 |
| RVD2 | RVD2-d5 | 4,95 | - | 375,40→175,20 | 375,40→217,15 | 23 | 22 | 14 | 1000 |
| LXA_4_ | LTB_4_-d4 | 5,21 | - | 351,50→115,05 | 351,50→217,15 | 19 | 14 | 14 | 500 |
| RVD1 | RVD2-d5 | 5,31 | - | 375,40→141,10 | 375,40→217,15 | 21 | 14 | 11 | 100 |
| RVD4 | RVD2-d5 | 5,77 | - | 375,40→101,05 | 375,40→357,30 | 21 | 21 | 13 | 50 |
| 5,15-DiHEPE (RVE4) | RVD2-d5 | 6,15 | - | 333,20→315,25 | 333,20→271,30 | 16 | 10 | 10 | 100 |
| 7,14-DiHDHA (Maresin 1) | RVD2-d5 | 6,90 | - | 359,40→177,25 | 359,40→93,15 | 20 | 16 | 10 | 500 |
| 10,17-DiHDHA (PDX) | RVD2-d5 | 6,99 | - | 359,30→153,15 | 359,30→206,25 | 20 | 17 | 19 | 100 |
| RVD5 | RVD2-d5 | 7,00 | - | 359,40→199,25 | 359,40→279,35 | 20 | 16 | 16 | 50 |
| 13,14-DiHDHA (Maresin 2) | RVD2-d5 | 7,66 | - | 359,40→221,05 | 359,40→232,25 | 12 | 12 | 18 | 100 |
| 20-COOH-LTB_4_ | LTB_4_-d4 | 3,59 | - | 365,00→347,25 | 365,00→195,20 | 11 | 18 | 19 | 500 |
| 20-OH-LTB_4_ | LTB_4_-d4 | 3,71 | - | 351,10→195,15 | 351,10→333,25 | 18 | 18 | 11 | 100 |
| LTB_5_ | LTB_4_-d4 | 6,12 | - | 333,40→195,25 | 333,40→315,10 | 11 | 14 | 11 | 100 |
| LTB_4_-d4 |  | 6,92 | - | 339,30→197,20 | 339,30→321,40 | 18 | 16 | 15 |  |
| 5,12-DiHETE | LTB_4_-d4 | 7,23 | - | 334,99→195,10 | 334,99→317,15 | 16 | 16 | 15 | 50 |
| LTB_4_ | LTB_4_-d4 | 6,94 | - | 335,30→195,25 | 335,30→317,30 | 18 | 16 | 11 | 50 |
| 12-oxo-LTB_4_ | LTB_4_-d4 | 7,51 | - | 333,40→179,20 | 333,40→128,90 | 19 | 14 | 14 | 50 |
| LTB_3_ | LTB_4_-d4 | 7,74 | - | 337,40→195,20 | 337,40→319,20 | 12 | 16 | 11 | 50 |
| DGLA | AA-d8 | 13,43 | - | 305,05→261,10 | 305,05→287,05 | 16 | 15 | 20 | 500 |

^a^Q1/Q3 and collision energy values refer to the quantifier and not the qualifier

^b^Lowest limit of quantification representing the lowest concentration of our calibration curve for which the signal-to-noise ratio was greater than 5.
